# Nanotechnology: A novel tool to enhance the bioavailability of micronutrients

**DOI:** 10.1002/fsn3.2311

**Published:** 2021-05-04

**Authors:** Rizwan Arshad, Lubaba Gulshad, Iahtisham‐Ul‐ Haq, Muhammad Adil Farooq, Ammar Al‐Farga, Rabia Siddique, Muhammad Faisal Manzoor, Emad Karrar

**Affiliations:** ^1^ University Institute of Diet and Nutritional Sciences The University of Lahore, Gujrat Campus Gujrat Pakistan; ^2^ School of Food and Nutrition Faculty of Allied Health Sciences Minhaj University Lahore Pakistan; ^3^ School of Food and Biological Engineering Jiangsu University Zhenjiang China; ^4^ Department of Food Science and Technology Khwaja Fareed University of Engineering and Information Technology Rahim Yar Khan Pakistan; ^5^ Department of Biochemistry College of Sciences University of Jeddah Jeddah Saudi Arabia; ^6^ Department of Chemistry Government College University Faisalabad Faisalabad Pakistan; ^7^ Department of Food Engineering and Technology Faculty of Engineering and Technology University of Gezira Wad Medani Sudan

**Keywords:** bioavailability, micronutrient, nanotechnology, novel tool

## Abstract

Nanotechnology has revolutionized the field of food systems, diagnostics, therapeutics, pharmaceuticals, the agriculture sector, and nutraceuticals. Nanoparticles are playing important role in giving the solution to enhance bioavailability of oral delivery of bioactive compounds. This review revealed that nanoparticles can improve the bioavailability of micronutrients, for example, vitamin B_12_, vitamin A, folic acid, and iron. However, toxicity associated with nanoparticle‐based delivery systems is still a major concern after ingestion of nano‐based supplements. The mode of the mechanism of nanomaterial along with bioactive components in different physiological conditions of the human body is also a major gap in the field of nanoceuticals. In the future, more evidence‐based clinical investigations are needed to confirm the exact approach to physiological changes in the human body.

## Graphical abstract

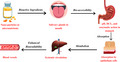

## 1 INTRODUCTION

Nanotechnology is modernizing the whole food chain from production to processing, storage, and consumption. Scientific reports suggest that nanotechnology can improve the thermal stability, water solubility, and oral bioavailability of nutrients (Ndlovu et al., 2020). Nanoparticles (NPs) for the delivery of edibles are biologically consistent tiny materials that vary in size from 1 to 100 nm and are produced through different methods. They have significant chemical and physical characteristics such as solubility, color, strength, infusibility, and high‐surface‐to‐volume ratio (Priyanka, 2018). These properties of NPs provide various valuable applications in different fields such as tissue engineering, cell therapy, drug delivery, diagnostic tools, biomaterials, and signaling molecules (Jain, 2020; Sajid et al., 2020).

Nanobiotechnology may assist in passing the barriers in the human body for targeted drug delivery to directed organs, for example, crossing the blood‐brain barrier to reach the brain. Nanomaterials found their applications not only in human wellbeing but also in food safety and quality (Jain, 2020). Recently, many organizations, researchers, and industries are adopting unique methods that have important applications of NPs in food technology (Dasgupta et al., 2015). Nanotechnology is being considered as a ray of hope for the treatment of the ongoing COVID‐19 outbreak. It is considered that chloroquine, an approved malaria drug may help in nano‐medicine investigation for the potential treatment of the COVID‐19 (Tony et al., 2020).

Functional food components such as vitamins, phytochemicals, minerals, and antioxidants are necessary for optimal human health and disease prevention. Nutraceuticals’ terms are a fusion of pharmaceutical and nutrition, which are derived from or any part of food that provides physiological, preventive, or therapeutic significance beyond the basic nutritional requirements (Yao et al., 2015). Worldwide, nutrition awareness and escalation in the rate of morbidity due to chronic diseases have developed a huge demand for dietary supplements. According to Prasad (2016), the global demand for nutraceuticals is dramatically expected to reach $302.307 million by 2022 which is foreseen based on a growth rate of 7.04% annually from 2016 to 2020. Moreover, it has been challenging to incorporate these nutraceuticals into food because they have chemical instability, undesirable flavor, and poor solubility characteristics (McClements et al., 2015). Various formulation tactics have been proposed to overcome the poor solubility but their benefits are limited by the possible interaction of excipients employed in preparation.

On the other hand, nanoparticle technology has received extensive acceptance as it includes the use of a minimum quantity of excipients to enhance the solubility of micronutrients (Charoo et al., 2019). The solution to these problems can also be delivered by encapsulating the bioactive ingredients in nanoparticle‐based delivery systems (Jafari & McClements, 2017). However, encapsulation of different micronutrients requires precise and appropriate nanoscale delivery systems depending on the properties as well as nature of the micronutrient (Joye et al., 2014). The objective of this review is to highlight the role of nanotechnology in improving the bioavailability of micronutrients through targeted delivery systems.

## 2 REVOLUTION OF NANOTECHNOLOGY IN THE FIELD OF NUTRITION

Nanotechnology has revolutionized the world and contributed to various fields including pharmacy, food processing industries, agriculture sectors, and nutrition (Nile et al., 2020). In the past few decades, research in the field of nutrition incorporating nano‐science has grown dynamically and stimulated a strong urge for targeted delivery of micronutrients (Dudefoi et al., 2017). Nano‐capsules are being used for the increased delivery of drugs and micronutrients (vitamins & minerals) in the body (Koo et al., 2005; McClements, 2020; Yan & Gilbert, 2004). In different methods, nano‐composite, nano‐structuration, and nano‐emulsification are used to encapsulate the materials in miniature forms to deliver bioactive compounds more efficiently. Encapsulated bioactive constituents (e.g., flavonoids and vitamins) can be formulated with polymeric nanomaterials for the protected nutrient delivery (Singh et al., 2017).

Nutrition‐be‐nanotech and nanoceuticals are names of some commercial supplements. Vitamin spray‐dispersed nano‐droplets are considered for improved absorption of nutrients, for example, iron, curcumin, and folic acid. Nano‐sized powders are also used for enhancing the absorption of nutrients as nano‐cochleate. These are revealed to be an effective tool for nutrient distribution to cells without affecting the taste and color of the food products. The supplement production mostly involves encapsulation techniques where the desirable probiotics and other products are directed into the human body with the help of Zn and Fe nano‐structured capsules. The NPs in food supplements are more active than commonly used supplements because they respond more effectively with human cells due to their special size (Koo et al., 2005; McClements et al., 2015).

Nano‐capsules are adapted as carriers for antioxidants, essential oils, flavors, coenzyme Q_10_, vitamins, phytochemicals, and minerals to improve their bioavailability in the human system (Ognik et al., 2016). The encapsulation of polyphenols with NPs may prevent any oxidative actions and providing them with an acceptable taste (Heller, 2006). In the food industry, liposomal nano‐vesicles have found applications for the supply and encapsulation of nutrients, enzymes, and antimicrobial compounds (Singh et al., 2017; Wen et al., 2006).

The role of nanotechnology is also assumed to improve the characteristics of bioactive particles in spices and herbs by enhancing their bioavailability, water solubility, and antioxidant properties enabling the active ingredients to dissolve homogeneously (Samah et al., 2017). The nano‐materials are considered to increase the bioavailability of important phytochemicals, for example, genistein and curcumin (Yen et al., 2010; Zhang et al., 2008). Moreover, nano‐nutraceuticals are available as dietary supplements, herbal products, and bioactive particles in nano‐formulations (He et al., 2019).

## 3 MICRONUTRIENT BIOAVAILABILITY

**FIGURE 1:**
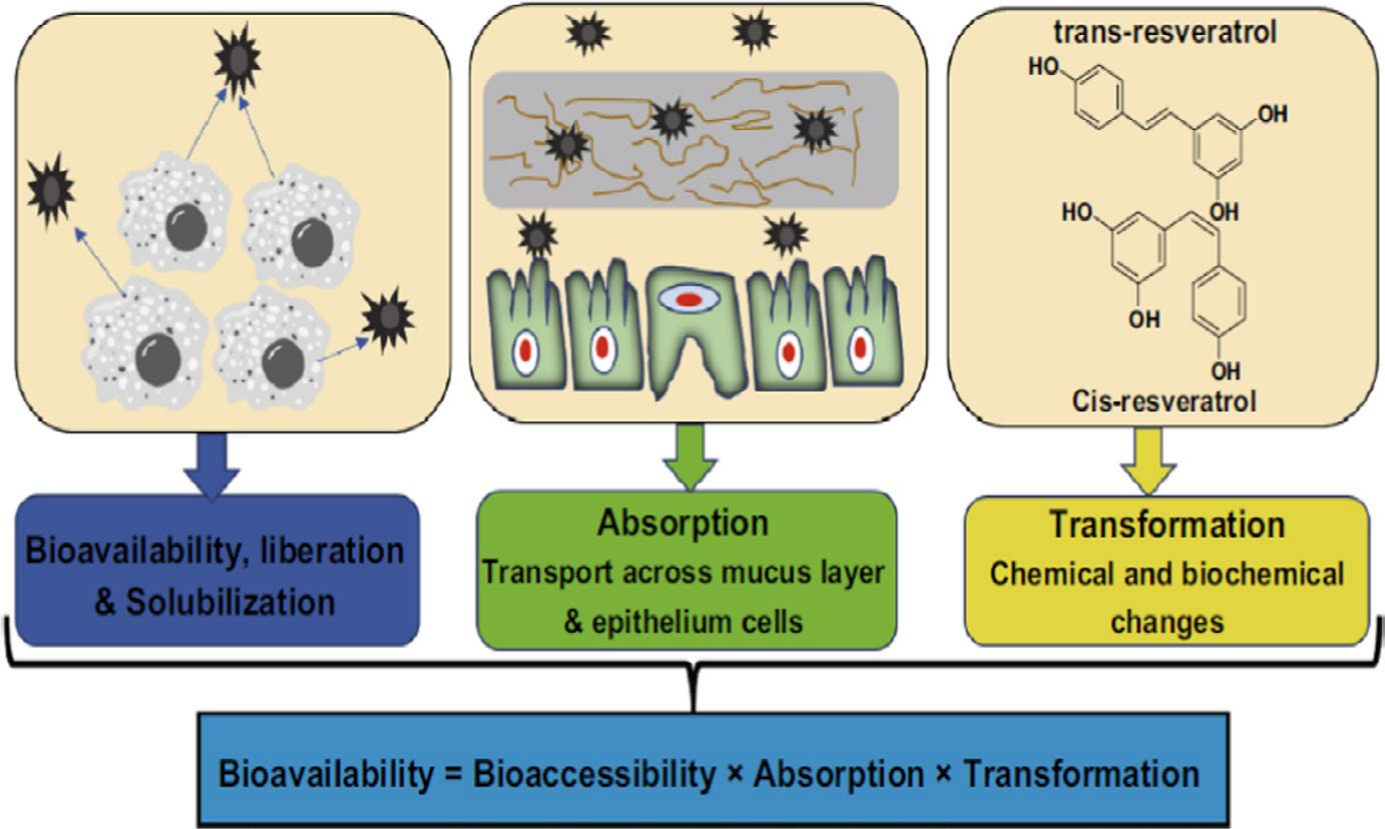
Mechanism of micronutrient bioavailability in the human body

According to Jafari and McClements (2017), the bioavailability of a nutrient is the fraction of ingested bioactive ingredient which is absorbed and consequently used for the essential physiological functions of the body. The bioavailability of bioactive compounds such as vitamins (A, D, and E), carotenoids, curcumin, conjugated linoleic acids, omega‐3 fatty acids, and coenzyme Q_10_ reduces after oral ingestion (Zhang & McClements, 2016). It happens due to physiological and physiochemical factors such as bioavailability, absorption, and transformation (Figure 1).

Yadav (2017) proposed the low bioavailability, stability, and solubility of bioactive molecules can be improved with the assistance of nanotechnology especially with nano‐formulations. Many biologically active particles utilized in dealing with diseases are hydrophobic with limited bioavailability. Nanotechnology‐based delivery systems are formulated to improve the targeted delivery of such nutrients. NPs are also prepared from natural food‐grade macro‐ingredients such as proteins, polysaccharides, lipids, and phospholipids, thus delivering no toxic effects. Nano‐emulsions are identified as numerous combinations of food‐grade ingredients such as lipid core along with protein shell (Kumar, 2000).

The efficacy, stability, and utilization of micronutrients can be improved with the assistance of food‐grade NPs via their encapsulating characteristic (Hildeliza et al., 2010). Different methods have been applied for the effective delivery of nutraceuticals. Nano‐fibers, nano‐sheets, fullerenes, nano‐whiskers, and nanotubes are disseminated through several carriers like liposomes, solid lipid NPs (SLNs), cubosomes, monolayers biopolymeric NPs, microemulsions, nano‐sensors, microemulsions, and fibers (Cushen et al., 2012).

**FIGURE 2:**
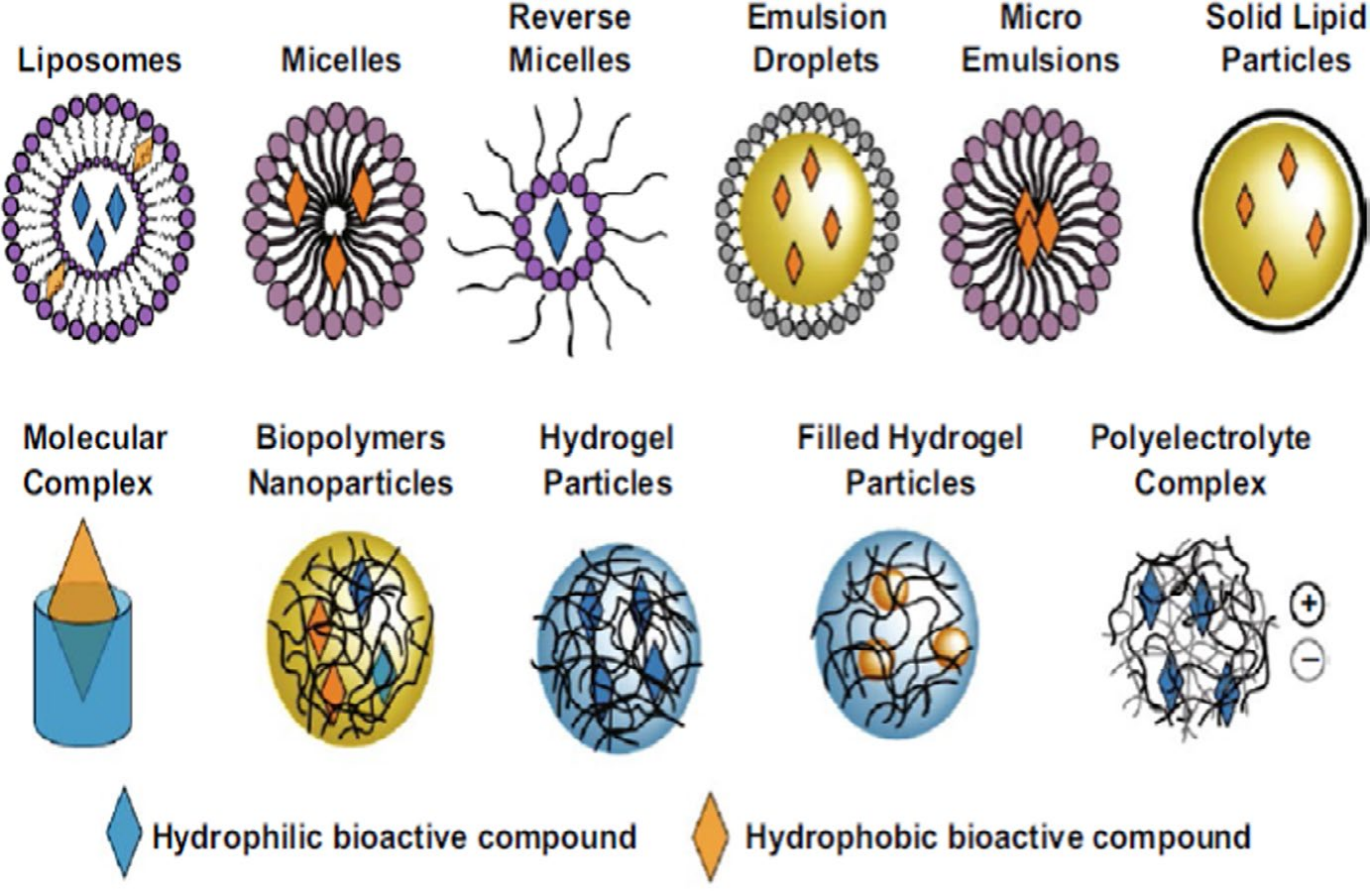
Different nano‐based delivery systems to improve the bioavailability of encapsulated micronutrients

McClements proposed that a successful colloidal delivery system must be designed precisely for a specific application depending on the nature of the functional particle and the end product (McClements, 2020). However, precisely designed excipient foods are used to improve the bio‐accessibility, absorption, and transformation of bioactive substances in the gastrointestinal tract (GIT). Many NPs based delivery systems are used to increase the bioavailability with appropriate encapsulation of micronutrients (hydrophilic and hydrophobic) as depicted in Figure 2 (McClements & Xiao, 2014).

## 4 STABILITY OF MICRONUTRIENTS IN THE PRODUCT

The breakdown of a micronutrient present in a food product significantly relay on its physicochemical and molecular properties, as well as the food composition and storage conditions. Micronutrients may be vulnerable to enzymatic, physical, and chemical instability within a food product. Chemical variability includes changes in the molecular arrangement which may lead to considerable variation in nutritional attributes and physicochemical properties of the bioactive component. Oxidation, hydrolysis, isomerization, and reduction are the common causes of chemical breakdown of micronutrients and these reactions can be carried out by enzymes present within the food products (McClements, 2015; McClements et al., 2009). Physical instability is considered as an alteration in the locality of micronutrients such as phase variations (e.g., crystallization, polymorphic transitions, or melting), gravitational separation, and aggregation. Whereas, principal degradation mechanisms and key factors (i.e., pH, temperature, and water activity) are critically significant to recognize for a specific micronutrient (Joye et al., 2014; Manzoor et al., 2019).

NPs may be layered with materials that inhibit the dissemination of one or more reactants into the particles. According to Matalanis et al. (2011), the degree of lipid oxidation in oil‐in‐water suspensions can be decreased by embedding the lipid molecules within a micro gel surrounded by protein molecules (Matalanis et al., 2011; Zhang et al., 2013). For example, the rate of lipid oxidation is lower in fish oil droplets captured within casein pectin microgels as compared to their free form (Zhang et al., 2013). These microgels may limit oxidation via several mechanisms such as antioxidant activity, chelating transition metals, and inhibiting dissemination of reactant (McClements, 2015).

## 5 NUTRIENT ENHANCER AND INHIBITORS AFFECTING BIOAVAILABILITY

Bioactive components may interact with each other or their surrounding medium causes modification in bioavailability. Nutrient enhancers such as vitamin C for iron and vitamin D for calcium can improve nutrient absorption or prevent their respective inhibitors. For instance, vitamin C can boost the absorption of iron by two to three folds (Teucher et al., 2004). On the other side, inhibitors hinder nutrient absorption either by binding it to other substances or lowering the absorption of nutrients by rendering it insoluble. Phytic acid is a common example of a minerals inhibitor that is present in many plant foods and has a strong binding potential for calcium, zinc, and iron that ultimately reduces or hinders the bioavailability of micronutrients (Zhou et al., 2010). In this context, nanotechnology can provide a promising solution to reduce the chemical inhibitors of bioactive particles.

According to Sasaki et al. (2011), curcumin dispersed with colloidal NPs found to have higher absorption efficacy than the natural curcumin powder. A study revealed that the color degradation rate of beta‐carotene dissolved in nano‐emulsions was much slower in the presence of a chemical inhibitor because of its attraction to chelate transition elements (such as Fe^2+^) that usually favor oxidation (Qian et al., 2012). Moreover, the oral bioavailability of curcumin can be nine folds higher when administered with (piperine) absorption enhancer (Shaikh et al., 2009; Haq et al., 2020).

## 6 BIOAVAILABILITY AFTER INGESTION

Bioavailability is a key point in ensuring the bioefficacy of oral drugs or bioactive food components. It is influenced by the chemical breakdown of bioactive elements during processing, manufacture, storage, and transport as well as manifestations within the gastrointestinal tract (GIT). Liberation, absorption, distribution, metabolism, and elimination phases are the main stages involved in the bioavailability of bioactive components. The bioavailability (*F*) can be expressed by the following equation: (McClements, 2012).

$$F=F_{C}\times F_{B}\times F_{A}\times F_{M}$$

Where *F_C_* is part of a micronutrient that acts as an active ingredient in a food product when orally ingested. *F_B_* is considered as bio‐accessibility; a portion of a micronutrient discharged from food product and dissolved in GIT liquids. *F_A_* called absorption, is the fraction of a micronutrient transported through the intestinal cavity. *F_M_* is named as metabolism, part of a micronutrient that acts as a bioactive form after gastrointestinal (GIT)alterations, for example, metabolic changes via the enzymatic activity, liver metabolism, or systemic circulation (McClements & Xiao, 2014).

Therefore, the bioavailability (F) of bioactive components can be improved through developing a targeted delivery system. This aim can be achieved by manipulating the structure and composition of nano‐based delivery systems. Some findings have resulted in the effectiveness of encapsulated triglycerides inside the microgels (e.g., calcium alginate beads) that obstruct breakdown in the upper GIT. When these beads reach the intestinal colon must be assimilated by an enzymatic breakdown and thus, releasing the bioactive fatty acid (Li et al., 2011).

Biopolymer NPs and microgels prepared by dietary fibers may be capable of delivering not only several lipophilic bioactive components into the lumen but also remain intact within GIT. However, it may be essential to design a biopolymer NP that protects the micronutrient within the food product. When designing biopolymer NPs for controlled release systems, it is necessary to investigate them by using in vitro virtual GIT models and practical in vivo animal studies along with human trials (McClements, 2015).

### 6.1 Vitamin A

The nano‐materials for vitamin A can be designed by understanding the bioavailability and biological processes that regulate the absorption of vitamin A. The encapsulation of vitamin A with nano‐materials may have more bioavailability than that of free vitamin A. This hypothesis was validated when researchers observed enhanced bioavailability of carotenoids when consumed with nano‐materials than the natural form of carotenoids (Faulks & Southon, 2005).

The bioavailability of vitamin A can also be boosted by nano‐materials which can help paracellular transport of vitamins by varying the integrity of close junctions of NPs. Moreover, oral bioavailability can also be improved when lipophilic composites, carried in a paracellular manner are not available to metabolic actions of intracellular enzymes. Therefore, the oral bioavailability (*F*) of encapsulated vitamin A with NPs can be defined by the following equation:

$$F=F_{B}\times F_{A}\times F_{M}$$

Here, *F_B_* is the percentage of consumed vitamin A that remains protected in the upper GIT and discharges from the nano‐materials into the colon, thus becoming bioavailable to enterocytes for absorption. *F_A_* is the percentage of bioavailable vitamin that extends into the portal blood circulation. *F_M_* is the portion of absorbed vitamin A that lasts in its active form following the first‐pass metabolism of the liver and GIT. Nanotechnology has successfully found its application in improving the oral bioavailability of vitamin A by protecting the physiochemical changes such as temperature, moisture, oxidation, and pH (Maurya & Aggarwal, 2017).

### 6.2 Vitamin B_12_

Protein lipid composite NPs containing three‐layer assembly (phospholipid, protein, and tocopherol layer) with an inner hydrophilic portion have been established recently as a transport system for hydrophilic bioactive elements. These improved NPs enhanced the uptake efficacy of vitamin B12 over 20 times (Yao et al., 2015). Vitamin B_12_ is absorbed in the terminal ileum with the help of an intrinsic factor that is produced by parietal cells in the stomach. Moreover, absorbed vitamin B_12_ is utilized as a cofactor for enzymes that are used in the synthesis of fatty acid, DNA, and myelin (Ankar & Kumar, 2019). While the in vivo findings indicated that the altered NPs could enhance a vitamin B12 insufficiency in a rat model more effectively than a free vitamin supplement (Liu et al., 2019). However, extensive research is needed to find a realistic approach to the increased bioavailability of vitamin B_12_ with nanotechnology.

### 6.3 Folic acid

According to Penalva et al. (2015) zein, NPs offer sufficient properties for oral targeted delivery of nutrients. When these NPs were orally administered, they remain confined within the GIT and protect the mucus layer of the jejunum. The encapsulation of folic acid with zein NPs enhanced its relatively oral bioavailability about 2 folds compared with an aqueous suspension of vitamin B_9_. This fact can be associated with the capacity of these particular NPs to affect the intestinal mucosa and establish the mucoadhesive associations (Penalva et al., 2015). However, there is a paucity of data in the clinical trials and about the effects of nanotechnology on the bioavailability of folic acid.

### 6.4 Iron

The preparation of iron with solid lipid NPs can enhance bioavailability by more than fourfold compared to the commercial tablets (Hosney et al., 2015). Katuwavila et al. (2016) demonstrated the production of ferrous (Fe^2+^) loaded with alginate NPs and its significant findings as a targeted system for oral iron consumption. Moreover, effective loading of ferrous (Fe^2+^) with alginate NPs was confirmed by using Fourier transform infrared spectroscopy and thermogravimetric analysis (Katuwavila et al., 2016). However, further in vivo studies and clinical trials are needed to confirm the efficacy of nano‐based delivery systems for the bioavailability of iron and other micronutrients.

## 7 TOXICITY OF NANOPARTICLES

Besides many significant applications of nanotechnology, some toxicities are associated with NPs (Ibrahim, 2013). The benefits of NPs include small size, great capacity, and high reactivity, which may be fatal by causing cellular lethal effects. NPs may cause the potential for adverse human effects due to their early appreciation in biotechnology via extended exposure in GIT. One of the common NPs toxicities is the ability to gather nearby the protein concentration that can be influenced by particle size, shape, and surface. Due to this unique binding capability, some nano‐materials produce adverse biotic consequences via protein fibrillation, unfolding, loss of enzymatic activity, and thiol crosslinking (Bahadar et al., 2016).

Moreover, NPs are readily bioavailable but their increased surface area can result in different toxicities. The discharge of lethal ions is another premise when the thermodynamic characteristics of substances may favor NPs discharge in a biological system (Xia et al., 2008). Moreover, nano‐structures established for curative tenacities are in very primary stages and the lasting adverse effects on human health need advanced research. However, a few numbers of studies are being done to decrease the toxicity of synthesized nano‐structures but still, there are crucial concerns regarding the potentials of NPs (Agrawal, 2016; Lambadi, 2015). The production, handling, and storage of nano‐structures may result in loss of efficacy and increase the risk of toxicities which are necessary to be investigated in detail.

## 8 FUTURE PERSPECTIVE AND TRENDS

The applications of nanotechnology can provide new insight into the fields of medicine, biological treatment, nutrition, food science, and biochemistry (Perez‐Esteve et al., 2016). However, there is still a long way to go for claims and applications of nano‐fortificants (Gallocchio et al., 2015). The safety concerns of nano‐materials are a prerequisite to be resolved because there are huge gaps in understanding the toxicities of NPs. However, it is the need of an hour to study the interaction of nano‐materials with food and their fate after ingestion. The key issues associated with “nano‐labeling” on the food supplements are also needed to take into consideration (Knijnenburg et al., 2019).

Moreover, NPs are being synthesized globally, but only a small number of countries have formulated the approved guidelines for the application of nanotechnology in the food industry. Regarding the effective delivery of micronutrients, insufficient scientific evidence on nanotechnology has caused complexities in giving definite conclusions, while NPs have a significant role in prospect expansion of curative and preventive applications for the targeted delivery of micronutrients (Nile et al., 2020).

## 9 CONCLUSIONS

Nanotechnology plays a dynamic role in improving the bioavailability of micronutrients through targeted delivery systems. Due to the significant thermodynamic properties of NPs, oral delivery of bioactive compounds can be enhanced. However, the research and findings by different researchers are supporting the increased bioavailability of micronutrients. The toxicity of NPs is questionable which can be problematic after ingestion of nano‐based supplements. Besides this, clinical findings and human‐based studies are still lacking in the literature to give a clear statement about the improved bioavailability of each of the essential micronutrients.

## AUTHOR CONTRIBUTIONS

**Rizwan Arshad:** Conceptualization (equal); Visualization (equal). **Lubaba Gulshad:** Software (equal); Writing‐original draft (equal). **Iahtisham Ul Haq:** Writing‐review & editing (equal). **adil farooq:** Writing‐review & editing (equal). **Ammar Al‐Farga:** Software (equal); Visualization (equal). **Rabia Siddique:** Writing‐original draft (equal). **Muhammad Faisal Manzoor:** Investigation (equal); Writing‐review & editing (equal). **Emad Karrar:** Writing‐review & editing (equal).

## Data Availability

The data used to support the findings of this study are available with the corresponding author upon request.

## References

[fsn32311-bib-0001] Agrawal, P. (2016). Potential prospects of future medicine: Nano medicine. Journal of Pharmacovigilance, 4(1), 1000–1149. 10.4172/2329-6887.1000e149

[fsn32311-bib-0002] Ankar, A. , & Kumar, A. (2019). Vitamin B12 deficiency (cobalamin). In StatPearls [Internet]. StatPearls Publishing. 28722952

[fsn32311-bib-0003] Bahadar, H. , Maqbool, F. , Niaz, K. , & Abdollahi, M. (2016). Toxicity of nanoparticles and an overview of current experimental models. Iranian Biomedical Journal, 20, 1–11. 10.7508/ibj.2016.01.001 26286636 PMC4689276

[fsn32311-bib-0004] Charoo, N. A. , Rahman, Z. , & Khan, M. A. (2019). Nanoparticles for improvement in oral bioavailability. In A. M. Grumezescu (Ed.), Nanoarchitectonics in Biomedicine (pp. 371–410). William Andrew Publishing.

[fsn32311-bib-0005] Cushen, M. , Kerry, J. , Morris, M. , Cruz‐Romero, M. , & Cummins, E. (2012). Nanotechnologies in the food industry‐recent developments, risks and regulation. Trends in Food Science Technology, 24, 30–46. 10.1016/JTIFS.2011.10.006

[fsn32311-bib-0006] Dasgupta, N. , Ranjan, S. , Mundekkad, D. , Ramalingam, C. , Shanker, R. , & Kumar, A. (2015). Nanotechnology in agro food: From field to plate. Food Research International, 69, 381–400. 10.1016/j.foodres.2015.01.005

[fsn32311-bib-0007] Dudefoi, W. , Villares, A. , Peyron, S. , Moreau, C. , Ropers, M. H. , Gontard, N. , & Cathala, B. (2017). Nanoscience and nanotechnologies for biobased materials, packaging and food applications: New opportunities and concerns. Innovative Food Science and Emerging Technologies, 46, 107–121. 10.1016/j.ifset.2017.09.007

[fsn32311-bib-0008] Faulks, R. M. , & Southon, S. (2005). Challenges to understanding and measuring carotenoid bioavailability. Biochimica et Biophysica Acta, 1740(2), 95–100. 10.1016/j.bbadis.2004.11.012 15949674

[fsn32311-bib-0009] Gallocchio, F. , Belluco, S. , & Ricci, A. (2015). Nanotechnology and food: Brief overview of the current scenario. Procedia Food Science, 5, 85–88. 10.1016/j.profoo.2015.09.022

[fsn32311-bib-0010] Haq, I. U. , Imran, M. , Nadeem, M. , Tufail, T. , Tanweer, A. G. , & Mubarak, M. S. (2020). Piperine: A review of its biological effects. Phytotherapy Research, 35(2), 680–700. 10.1002/ptr.6855 32929825

[fsn32311-bib-0011] He, X. , Deng, H. , & Hwang, H. (2019). The current application of nanotechnology in food and agriculture. Journal of Food and Drug Analysis, 27, 1–21. 10.1016/J.JFDA.2018.12.002 30648562 PMC9298627

[fsn32311-bib-0012] Heller, L. (2006). Flavor firm uses nanotechnology for new ingredient solutions. Food Navigator News. http://www.foodnavigator‐usa.com/Suppliers2/Flavor‐firm‐usesnanotechnology‐for‐new‐ingredient‐solutions

[fsn32311-bib-0013] Hildeliza, Q. B. , Chanona‐pe, J. , Jose, L. S. M. , Gutie, G. F. , & Jimene, A. (2010). Nano‐encapsulation: A new trend in food engineering processing. Food Engineering Reviews, 2, 39–50. 10.1007/s12393-009-9012-6

[fsn32311-bib-0014] Hosney, K. M. , Banjar, Z. M. , Hariri, A. H. , & Hassan, A. H. (2015). Solid lipid nanoparticles loaded with iron to overcome barriers for treatment of iron deficiency anemia. Drug Design, Development and Therapy, 9, 313. 10.2147/DDDT.S77702 PMC4293289 25609917

[fsn32311-bib-0015] Ibrahim, K. S. (2013). Carbon nanotubes‐properties and applications: A review. Carbon Letters, 14, 131–144. 10.5714/CL.2013.14.3.131

[fsn32311-bib-0016] Jafari, S. M. , & McClements, D. J. (2017). Nanotechnology approaches for increasing nutrient bioavailability. Advances in Food and Nutrition Research, 81, 1–30. 10.1016/bs.afnr.2016.12.008 28317602

[fsn32311-bib-0017] Jain, K. K. (2020). Role of nanobiotechnology in drug delivery. In K. Jain (Eds.), Drug delivery systems, methods in molecular biology 2059 (pp. 55‐73). Humana. 10.1007/978-1-4939-9798-5-2 31435915

[fsn32311-bib-0018] Joye, I. J. , Davidov‐Pardo, G. , & McClements, D. J. (2014). Nanotechnology for increased micronutrient bioavailability. Trends in Food Science & Technology, 40(2), 168–182. 10.1016/j.tifs.2014.08.006

[fsn32311-bib-0019] Katuwavila, N. P. , Perera, A. D. , Dahanayake, D. , Karunaratne, V. , Amaratunga, G. A. , & Karunaratne, D. N. (2016). Alginate nanoparticles protect ferrous from oxidation: Potential iron delivery system. International Journal of Pharmaceutics, 513(1–2):404–419. 27659860 10.1016/j.ijpharm.2016.09.053

[fsn32311-bib-0020] Knijnenburg, J. T. , Posavec, L. , & Teleki, A. (2019). Nanostructured minerals and vitamins for food fortification and food supplementation. In A. L. Rubio , M. M. Sanz , M. J. F. Rovira , & L. G. Gómez‐Mascaraque (Eds.), Nanomaterials for Food Applications (pp. 63–98). Elsevier.

[fsn32311-bib-0021] Koo, O. M. , Rubinstein, I. , & Onyuksel, H. (2005). Role of nanotechnology in targeted drug delivery and imaging: A concise review. Nanomedicine: Nanotechnology, Biology and Medicine, 1, 193–212. 10.1016/j.nano.2005.06.004 17292079

[fsn32311-bib-0022] Kumar, M. N. R. (2000). Nano and microparticles as controlled drug delivery devices. Journal of Pharmacy and Pharmaceutical Sciences, 3(2), 234–258.PMID: 10994037. 10994037

[fsn32311-bib-0023] Lambadi, P. R. (2015). Facilebiofunctionalization of silver nanoparticles for enhanced antibacterial properties, endotoxin removal, and biofilm control. International Journal of Nanomedicine, 10, 2155–2171. 10.2147/IJN.S72923 25834431 PMC4370915

[fsn32311-bib-0024] Li, Y. , Hu, M. , Du, Y. M. , Xiao, H. , & McClements, D. J. (2011). Control of lipase digestibility of emulsified lipids by encapsulation within calcium alginate beads. Food Hydrocolloids, 25, 122–130. 10.1016/j.foodhyd.2010.06.00

[fsn32311-bib-0025] Liu, G. , Yang, J. , Wang, Y. , Liu, X. , & Chen, L. (2019). Protein‐lipid composite nanoparticles for the oral delivery of vitamin B12: Impact of protein succinylation on nanoparticle physicochemical and biological properties. Food Hydrocolloids, 92, 189–197. 10.1016/j.foodhyd.2018.12.020

[fsn32311-bib-0026] Manzoor, M. F. , Ahmad, N. , Ahmed, Z. , Siddique, R. , Zeng, X.‐A. , Rahaman, A. , Muhammad Aadil, R. , & Wahab, A. (2019). Novel extraction techniques and pharmaceutical activities of luteolin and its derivatives. Journal of Food Biochemistry, 43(9), e12974. 10.1111/jfbc.12974 31489656

[fsn32311-bib-0027] Matalanis, A. , Jones, O. G. , & McClements, D. J. (2011). Structured biopolymer‐based delivery systems for encapsulation, protection, and release of lipophilic compounds. Food Hydrocolloids, 25, 1865–1880. 10.1016/j.foodhyd.2011.04.014

[fsn32311-bib-0028] Maurya, V. K. , & Aggarwal, M. (2017). Enhancing bio‐availability of vitamin D by Nano‐engineered based delivery systems‐an overview. International Journal of Current Microbiology and Applied Sciences, 6, 340–353. 10.20546/ijcmas.2017.607.040

[fsn32311-bib-0029] McClements, D. J. (2012). Advances in fabrication of emulsions with enhanced functionality using structural design principles. Current Opinion in Colloid & Interface Science, 17, 235–245. 10.1016/j.cocis.2012.06.002

[fsn32311-bib-0030] McClements, D. J. (2015). Nanoscale nutrient delivery systems for food applications: Improving bioactive dispersibility, stability, and bioavailability. Journal of Food Science, 80(7), N1602–N1611. 10.1111/1750-3841.12919 26073042

[fsn32311-bib-0031] McClements, D. J. (2020). Recent advances in the production and application of nano‐enabled bioactive food ingredients. Current Opinion in Food Science, 33, 85–90. 10.1016/j.cofs.2020.02.004

[fsn32311-bib-0032] McClements, D. J. , Decker, E. A. , Park, Y. , & Weiss, J. (2009). Structural design principles for delivery of bioactive components in nutraceuticals and functional foods. Critical Reviews in Food Science and Nutrition, 49(6), 577–606. 10.1080/10408390902841529 19484636

[fsn32311-bib-0033] McClements, D. J. , Li, F. , & Xiao, H. (2015). The nutraceutical bioavailability classification scheme: Classifying nutraceuticals according to factors limiting their oral bioavailability. Annual Review of. Food Science and Technology, 6, 299–327. 10.1146/annurev-food-032814-014043 25705933

[fsn32311-bib-0034] Mcclements, D. J. , & Xiao, H. (2014). Excipient foods: Designing food matrices that improve the oral bioavailability of pharmaceuticals and nutraceuticals. Food & Function, 5, 1307–1632. 10.1039/c4fo00100 24760211

[fsn32311-bib-0035] Ndlovu, N. , Mayaya, T. , Muitire, C. , & Munyengwa, N. (2020). Nanotechnology applications in crop production and food systems. International Journal of Plant Breeding and Crop Science, 7(1), 624–634.

[fsn32311-bib-0036] Nile, S. H. , Baskar, V. , Selvaraj, D. , Nile, A. , Xiao, J. , & Kai, G. (2020). Nanotechnologies in Food Science: Applications, Recent Trends, and Future Perspectives. Nano‐Micro Letters, 12(1), 45. 10.1007/s40820-020-0383-9 PMC7770847 34138283

[fsn32311-bib-0037] Ognik, K. , Cholewinska, E. , Czech, A. , Kozlwski, K. , Wlazlo, L. , Nowakowicz‐Dębek, B. , Szlązak, R. , & Tutaj, K. (2016). Effect of silver nanoparticles on the immune, redox and lipid status of chicken blood. Czech Journal of Animal Science, 61, 450–461. 10.17221/80/2015-CJAS

[fsn32311-bib-0038] Penalva, R. , Esparza, I. , González‐Navarro, C. J. , Quincoces, G. , Peñuelas, I. , & Irache, J. M. (2015). Zein nanoparticles for oral folic acid delivery. Journal of Drug Delivery Science and Technology, 30, 450–457. 10.1016/j.jddst.2015.06.012

[fsn32311-bib-0039] Perez‐Esteve, E. , Ruiz‐Rico, M. , De La Torre, C. , Villaescusa, L. A. , Sancen_on, F. , Marcos, M. D. , Amor_os, P. , Martínez‐Ma~nez, R. , & Barat, J. M. (2016). Encapsulation of folic acid in different silica porous supports: A comparative study. Food Chemistry, 196, 66–75. 26593466 10.1016/j.foodchem.2015.09.017

[fsn32311-bib-0040] Prasad, E. (2016). Nutraceuticals market‐ global opportunity analysis and industry forecast, 2014–2022. https://www.alliedmarketresearch.com/nutraceuticals‐market

[fsn32311-bib-0041] Priyanka, S. (2018). Nanotechnology in food preservation. Food Science Research Journal, 9(2), 441–447. 10.15740/HAS/FSRJ/9.2/441-447

[fsn32311-bib-0042] Qian, C. , Decker, E. A. , Xiao, H. , & McClements, D. J. (2012). Inhibition of beta‐carotene degradation in oil‐in‐water nanoemulsions: Influence of oil‐soluble and water‐soluble antioxidants. Food Chemistry, 135(3), 1036–1043. 10.1080/10408398.2016.1223599 22953821

[fsn32311-bib-0043] Sajid, A. , Manzoor, Q. , Sajid, A. , Imran, M. , Khalid, S. , Arshad, Z. , Saleem, A. , Haq, I. U. , & Aslam, F. (2020). Characterization and antibacterial properties of Eriobotrya japonica extract loaded silver‐nanoparticles. Current Bioactive Compounds, 10.2174/1573407216999201208203418

[fsn32311-bib-0044] Samah, N. A. , Mahmood, M. R. , & Muhamad, S. (2017). The role of nanotechnology application in antioxidant from herbs and spices for improving health and nutrition: A review. Selangor Science & Technology Review, 1(1), 13–17. http://sester.journals.unisel.edu.my/ojs/index.php/sester/article/view/19

[fsn32311-bib-0045] Sasaki, H. , Sunagawa, Y. , Takahashi, K. , Imaizumi, A. , Fukudu, H. , Hashimoto, T. , Wada, H. , Katanasaka, Y. , Kakeya, H. , Fujita, M. , Hasegawa, K. , & Morimoto, T. (2011). Innovative preparation of curcumin for improved oral bioavailability. Biological and Pharmaceutical Bulletin, 34(5), 660–665. 10.1248/bpb.34.660 21532153

[fsn32311-bib-0046] Shaikh, J. , Ankola, D. , Beniwal, V. , Singh, D. , & Kumar, M. R. (2009). Nanoparticle encapsulation improves oral bioavailability of curcumin by at least 9‐fold when compared to curcumin administered with piperine as absorption enhancer. European Journal of Pharmaceutical Sciences, 37(3‐4), 223–230. 19491009 10.1016/j.ejps.2009.02.019

[fsn32311-bib-0047] Singh, T. , Shukla, S. , Kumar, P. , Wahla, V. , Bajpai, V. K. , & Rather, I. A. (2017). Application of nanotechnology in food science: Perception and overview. Frontiers in Microbiology, 8, 1501. 10.3389/fmicb.2017.02517 28824605 PMC5545585

[fsn32311-bib-0048] Teucher, B. , Olivares, M. , & Cori, H. (2004). Enhancers of iron absorption: Ascorbic acid and other organic acids. International Journal for Vitamin and Nutrition Research, 74(6), 403–419. 10.1024/0300-9831.74.6.403 15743017

[fsn32311-bib-0049] Tony, Y. H. , Frieman, M. , & Wolfram, J. (2020). Insights from nanomedicine into chloroquine efficacy against COVID‐19. Nature Nanotechnology, 15, 247–249. 10.1038/s41565-020-0674-9 PMC7094976 32203437

[fsn32311-bib-0050] Wen, H. W. , DeCory, T. R. , Borejsza‐Wysocki, W. , & Durst, R. A. (2006). Investigation of Neutr Avidin‐tagged liposomal nanovesicles as universal detection reagents for bioanalytical assays. Talanta, 68, 1264–1272. 10.1016/j.talanta.2005.07.032 18970459

[fsn32311-bib-0051] Xia, T. , Kovochich, M. , Liong, M. , Mädler, L. , Gilbert, B. , Shi, H. , Yeh, J. I. , Zink, J. I. , & Nel, A. E. (2008). Comparison of the mechanism of toxicity of zinc oxide and cerium oxide nanoparticles based on dissolution and oxidative stress properties. ACS Nano, 2, 2121–2134. 10.1021/nn800511k 19206459 PMC3959800

[fsn32311-bib-0052] Yadav, S. K. (2017). Tissue science & engineering realizing the potential of nanotechnology for agriculture and food technology. Journal of Tissue Science & Engineering, 8, 8‐11. 10.4172/2157-7552.1000195

[fsn32311-bib-0053] Yan, S. S. , & Gilbert, J. M. (2004). Antimicrobial drug delivery in food animals and microbial food safety concerns: An overview of in vitro and in vivo factors potentially affecting the animal gut microflora. Advanced Drug Delivery Reviews, 56, 1497–1521. 10.1016/j.addr.2004.02.010 15191795

[fsn32311-bib-0054] Yao, M. , McClements, D. J. , & Xiao, H. (2015). Improving oral bioavailability of nutraceuticals by engineered nanoparticle‐based delivery systems. Current Opinion in Food Science, 2, 14–19. 10.1016/j.cofs.2014.12.005

[fsn32311-bib-0055] Yen, F. L. , Wu, T. H. , Tzeng, C. W. , Lin, L. T. , & Lin, C. C. (2010). Curcumin nanoparticles improve the physicochemical properties of curcumin and effectively enhance its antioxidant and antihepatoma activities. Journal of Agriculture Food Chemistry, 58, 7376–7382. 10.1021/jf100135h 20486686

[fsn32311-bib-0056] Zhang, R. , & McClements, D. J. (2016). Enhancing nutraceutical bioavailability by controlling the composition and structure of gastrointestinal contents: Emulsion‐based delivery and excipient systems. Food Structure, 10, 21–36. 10.1016/J.FOOSTR.2016.07.006

[fsn32311-bib-0057] Zhang, X. P. , Le, Y. , Wang, J. X. , Zhao, H. , & Chen, J. F. (2013). Resveratrol nanodispersion with high stability and dissolution rate. Lwt‐Food Science and Technology, 50, 622–628. 10.1016/j.lwt.2012.07.041

[fsn32311-bib-0058] Zhang, Y. , Yang, Y. , Tang, K. , Hu, X. , & Zou, G. (2008). Physicochemical characterization and antioxidant activity of Quercetin‐loaded Chitosa nanoparticles. Journal of Applied Polymer Science, 107, 891–897. 10.1002/app.26402

[fsn32311-bib-0059] Zhou, H. , Yue, Y. , Liu, G. , Li, Y. , Zhang, J. , Gong, Q. , Yan, Z. , & Duan, M. (2010). Preparation and characterization of a lecithin nanoemulsion as a topical delivery system. Nanoscale Research Letters, 5(1), 224–230. 10.1007/s11671-009-9469-5 PMC2894193 20652152

